# Can compliment and complaint data inform the care of individuals with chronic subdural haematoma (cSDH)?

**DOI:** 10.1136/bmjoq-2020-001246

**Published:** 2021-09-16

**Authors:** Katherine Jones, Benjamin Davies, Daniel J Stubbs, Alexander Komashie, Rowan M Burnstein, Peter Hutchinson, Thomas Santarius, Alexis J Joannides

**Affiliations:** 1 University of Cambridge School of Clinical Medicine, Cambridge, UK; 2 Academic Neurosurgery, University of Cambridge, Cambridge, UK; 3 University Division of Anaesthesia, University of Cambridge, Cambridge, UK; 4 The Healthcare Improvement Studies (THIS) Institute, University of Cambridge, Cambridge, Cambridgeshire, UK; 5 Neurocritical Care Department and Department of Anaesthesia, University of Cambridge, Cambridge, Cambridgeshire, UK

**Keywords:** health services research, quality improvement, patient satisfaction, quality measurement

## Abstract

**Objectives:**

To explore the frequency and nature of complaints and compliments reported to Patient Advice and Liaison (PALS) in individuals undergoing surgery for a chronic subdural haematoma (cSDH).

**Design:**

A retrospective study of PALS user interactions.

**Subjects:**

Individuals undergoing treatment for cSDH between 2014 and 2019.

**Methods:**

PALS referrals from patients with cSDH between 2014 and 2019 were identified. Case records were reviewed and data on the frequency, nature and factors leading up to the complaint were extracted and coded according to Healthcare Complaints Analysis Tool (HCAT).

**Results:**

Out of 531 patients identified, 25 (5%) had a PALS interaction, of which 15 (3%) were complaints and 10 (2%) were compliments. HCAT coding showed 8/15 (53%) of complaints were relationship problems, 6/15 (33%) a management problem and 1/15 (7%) other. Of the relationship problems, 6 (75%) were classed as problems with communication and 2 (25%) as a problem with listening. Of the compliments, 9/10 (90%) related to good clinical quality and 1/10 (10%) to staff–patient relationship. Patients were more likely to register a compliment than family members, who in turn were more likely to register a complaint (p<0.005). Complaints coded as a relationship problem had 2/8 (25%) submitted by a patient and 6/8 (75%) submitted by a relative.

**Conclusions:**

Using the HCAT, routinely collected PALS data can easily be coded to quantify and provide unique perspective on tertiary care, such as communication. It is readily suited to quality improvement and audit initiatives.

## Introduction

National Health Service (NHS) Hospitals in the UK must provide a Patient Advise and Liason Service (PALS) as a means of impartial mediation between the healthcare user and the provider. PALS interactions can be negative (complaints) or positive (compliments). Consequently, PALS data offer the potential to inform and monitor the extremes of substandard and excellent care. This information could be a key source of data to both inform and monitor service evaluation or quality improvement processes.

Previous analysis of complaints data has also shown its potential to capture unique perspectives.^1^ For example, one study identified that adverse events were more reliably reported by patients through PALS than staff through incident reporting systems,^2^ while another that patients often voice and identify issues through complaints, not otherwise captured within the formal medical record.^3^ Moreover, not only patients submit complaints—family, friends and staff can also raise concerns.^4^

In practice, complaint cases are typically dealt with on an individual basis. However, their aggregate analysis has the potential to offer pervasive insights into clinical care. While an extreme example, the opportunities to identify and change systemic issues that can be overlooked at the level of the individual were considered a significant contributor to failures at mid-Staffordshire NHS Foundation Trust, leading to neglect, substandard care and death.^5^ This aggregate approach would appear to align with patient wishes who submit complaints with the purpose of improving healthcare rather than for personal satisfaction or to trigger disciplinary action.^6^ To support and standardise the use of complaints and compliments in healthcare improvement, a taxonomic system called the Healthcare Complaints Analysis Tool (HCAT)^7^ was developed for complaints data with a subsequent adaptation proposed to incorporate compliment data.^8^

Patients receiving specialist care from a tertiary specialty often do so on a region wide basis in a so called ‘hub and spoke’ model, which can raise particular challenges in healthcare delivery. The utility of complaints data to inform care in this setting has not been previously reported. On this background, we sought to evaluate PALS data associated with care of a common neurosurgical condition, chronic subdural haematoma (cSDH).

A cSDH is an encapsulated collection of fluid, blood and blood degradation products^9^ that can form between the skull and the brain. Although it is often an incidental finding on imaging, neurological symptoms can arise due to compression of the brain. In this instance, surgical evacuation is performed. Patients with cSDH are typically triaged by their local healthcare provider, and those requiring treatment transferred to a neurosurgical centre, before returning to their local healthcare provider for rehabilitation if required.

Patients with cSDH pose an increasing challenge for neurosurgical care, as the condition typically occurs in elderly comorbid populations and, with well-documented changes in population demographics,^10^ these numbers may rise.^11^ Consequently, methods to monitor and optimise care delivery would be valuable.^12 13^ This can be a particular challenge for a service involving multiple healthcare providers or centres.^14^

This project sought to evaluate the application of the HCAT to a cohort of operated patients with cSDH, as an exemplar of its potential for quality improvement within neurosurgery and for tertiary services in general.

## Methods

### Setting

This was a single-centre, retrospective, study conducted of patients treated surgically for a cSDH between October 2014 and January 2019. The study centre provides neurosurgical services to a regional population of approximately 4 million people.^15^

### Case identification

cSDH cases were identified by screening a combination of theatre activity logs and our prospectively maintained referrals database. In total, 531 cases were identified. PALS interactions for Neurosurgery were then requested for the same time period, and cross-referenced using hospital numbers.

### Data extraction and analysis

PALS records included details of the nature of interaction (complaint or compliment), case summary, source of complaint (patient, family or staff member) and outcome. Within the study centre, each negative interaction was coded as either a concern or a complaint (‘Are you making a complaint, or are you raising a concern?’). These are both handled by PALS in the same manner. This distinction aims to remove a potential barrier to registering feedback, as some users consider a ‘complaint’ negative, when they only wish to provide constructive feedback. For this study, these were considered together as complaints.

**Figure 1:**
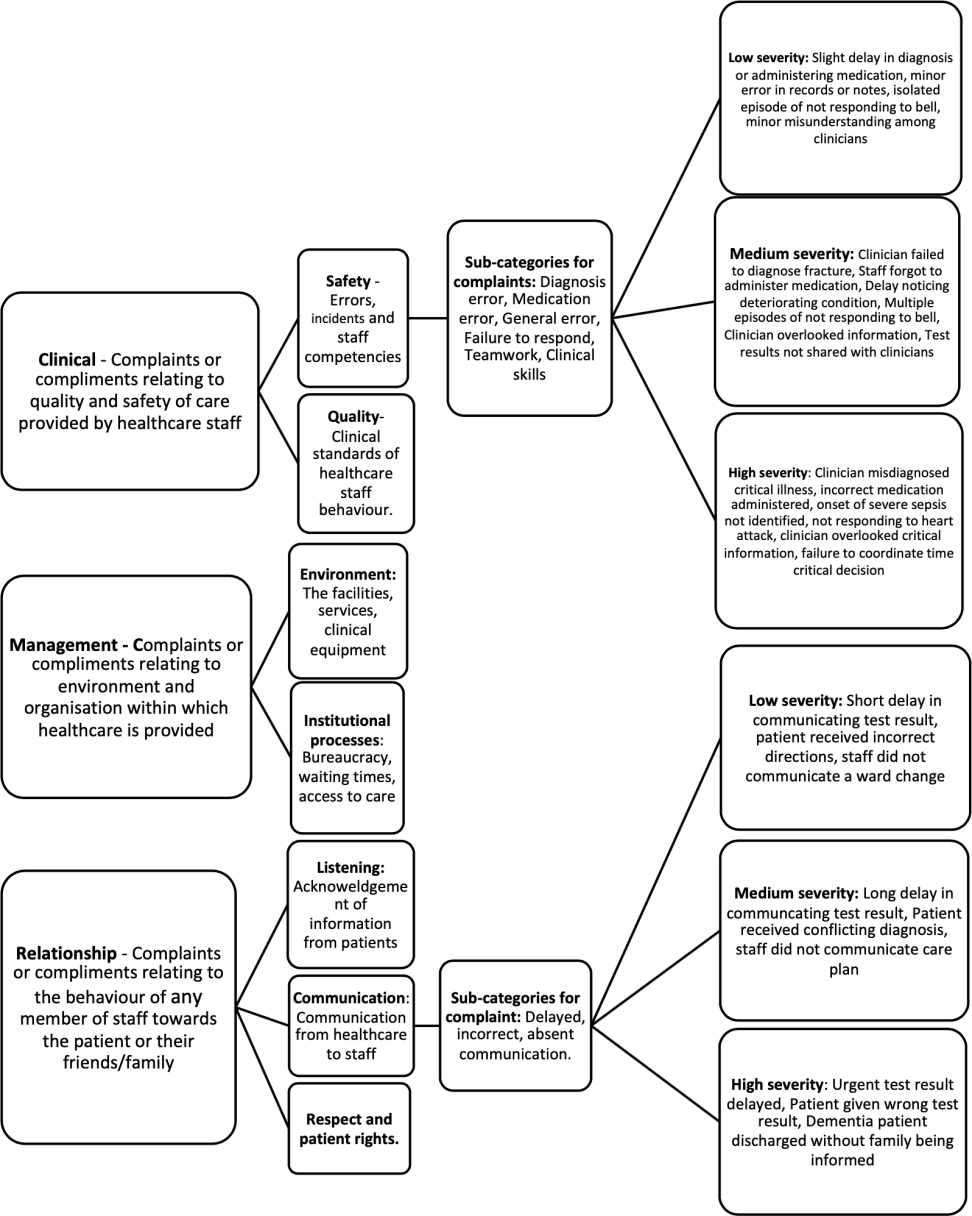
Overview of the Healthcare Complaints Analysis Tool (HCAT), including its adaption for compliments, from Gillespie *et al* (2016).

**Figure 2:**
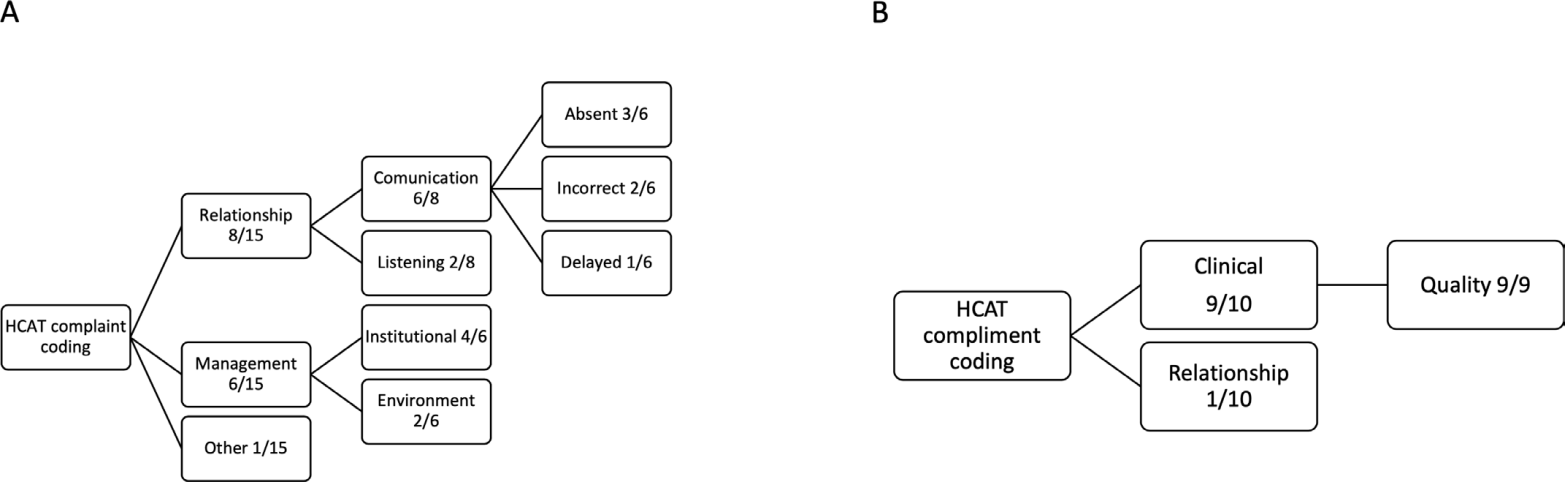
Frequency of complaints and compliments, across the Healthcare Complaints Analysis Tool (HCAT) domains and subdomains.

PALS data were categorised according to the HCAT coding algorithm (figure 1). Complaints were also coded by harm arisen as set out by Gillespie *et al*^16^ to emulate a risk and impact approach similar to the Quality Surveillance Information System (QSIS) incident stratification system. A modified HCAT coding was also used to analyse the compliments with a view to stratifying a model of good care (figure 2B). HCAT coding was completed independently by two authors (BMD and KJ) and inter-rater agreement compared using a Kappa statistic.^17^ Any conflicts were settled by mutual discussion.

In order to evaluate the role of PALS data against traditional performance indicators, clinical care metrics, included those previously identified in this series of patients^11^ and for cSDH in general by others^12 13^ were taken from hospital care records. This included age, gender, independence before admission, American Society of Anesthesiologists score, admission date, referral source (local vs referred), operation date, discharge date, whether they required a reoperation, anticoagulation status and occurrence of an in-hospital complication. In-hospital complications were as myocardial injury (troponin above 14 ng/L) or acute kidney injury (>50% rise in creatinine above baseline).^11^ Statistical testing was performed using Fisher’s exact test with significance determined as a p value of<0.05.

### Approvals

The project was approved as a retrospective evaluation of service by the hospitals quality, safety and improvement department^11^

### Patient and public involvement

This project explores the use of routinely captured information on patient experience to improve care. Patients, however, were not specifically consulted on the study design or its conduct. The results are being used to inform a national UK initiative, involving patient stakeholders, to optimise the care of people with cSDH called Improving Care in Elderly Neurosurgery Initiative. For more information, visit www.improving-care.in/chronic-subdural-haematoma.

## Results

A total of 531 patients underwent surgery for cSDH between October 2014 and January 2019, of which 25 (5%) had an interaction with PALS including 15 (3%) complaints and 10 (2%) compliments (online supplemental 1). All complaints had been raised as concerns with PALs.

10.1136/bmjoq-2020-001246.supp1 Supplementary data

### Performance of HCAT coding

The inter-rater agreement with application of the HCAT algorithm was high, with only 1 (4%) disagreement arising between reviewers (Kappa=0.9); study ID 2 (online supplemental 1)—‘… concerned about repatriation to local hospital due to concerns about care they had previously received there’. This was coded by the one review as ‘Clinical problem—Quality—clinical standards’ and the other as ‘Management problem—Institutional problem—accessing care’ due to an inability to control the safety or quality of another hospital. Quotations from individual cases used to inform HCAT categorisation are aggregated in online supplemental 2. While the majority of compliments pertained to overall care, complaints focused on specific aspects of care.

10.1136/bmjoq-2020-001246.supp2 Supplementary data

Figure 2A depicts the resolved categories. Of the complaints, 7/15 (47%) were coded low severity and 8/15 (53%) medium severity. No complaints were graded high severity and only one case of minimal harm was reported (online supplemental 1). Of 15, 8 (53%) of complaints were classified as being a relationship problem, 6/15 (33%) a management problem and 1/15 (7%) other. Of the relationship problems, 6 (75%) were classed as problems with communication and 2 (25%) as a problem with listening. The communication problem could be further categorised into an absence (3/6%–50%), incorrect (2/6%–33%) and delayed (1/6%–17%) communication. Of the complaints classed as problems with management, 4 (67%) were deemed institutional (including waiting times, transfer process and accessing care) and 2 (33%) as environmental problems (including facilities and accommodation). Of the compliments, 9/10 (90%) related to good clinical quality and 1/10 (10%) to staff–patient relationship (figure 2B).

### Comparison of compliments with complaints

On the basis compliments could be considered as idealised care and complaints suboptimal care, variables were compared between these groups. Table 1 depicts the distribution of PALS interactions in our study cohort against identified risk factors^11^ and highlighted that the only significant value was the source of the PALS interaction. Patients were more likely to register a compliment than family members, who in turn were more likely to register a complaint (p<0.005). Of the complaints coded as a management problem, 4/6 (67%) were submitted by relatives, 1/6 (17%) by a patient and 1/6 (17%) by a staff member. Complaints coded as a relationship problem had 2/8 (25%) submitted by a patient and 6/8 (75%) submitted by a relative.

**Table 1:** This table summarises the analysis of variables in relation to percentage of complaints and compliments

Variables		Study cohort (% of cohort)	Complaints (% of complaints)	Compliments (% of compliments)	P value (*<0.05)
N		25	15	10	
Median age (IQR)		79 (72–86)	79 (72–85)	81 (68–87)	0.94
Gender	Male	21 (84)	11 (73)	10 (100)	0.12
ASA	n with ASA ≥ 3	14 (56)	7 (47)	7 (70)	0.41
Source of PALS interaction	Patient	12 (48)	3 (20)	9 (90)	0.001^*^
	Other	13 (52)	12 (80)	1 (10)	
Referred from other hospital	Yes	16 (64)	10 (67)	6 (60)	1.0
Length of stay	>10 days	16 (64)	11 (73)	5 (50)	0.4
Admission to operation	>1 day	8 (32)	5 (33)	3 (30)	1.0
Re-operation	Yes	7 (28)	6 (40)	1 (10)	0.2
Anticoagulation	Yes	7 (28)	4 (27)	3 (30)	0.7
Complication (AKI or MI)	Yes	5 (20)	3 (20)	2 (20)	1.0

AKI, acute kidney injury; ASA, American Society of Anesthesiologists; cSDH, chronic subdural haematoma; MI, myocardial injury; PALS, Patient Advise and Liason Service.

**Figure 3:**
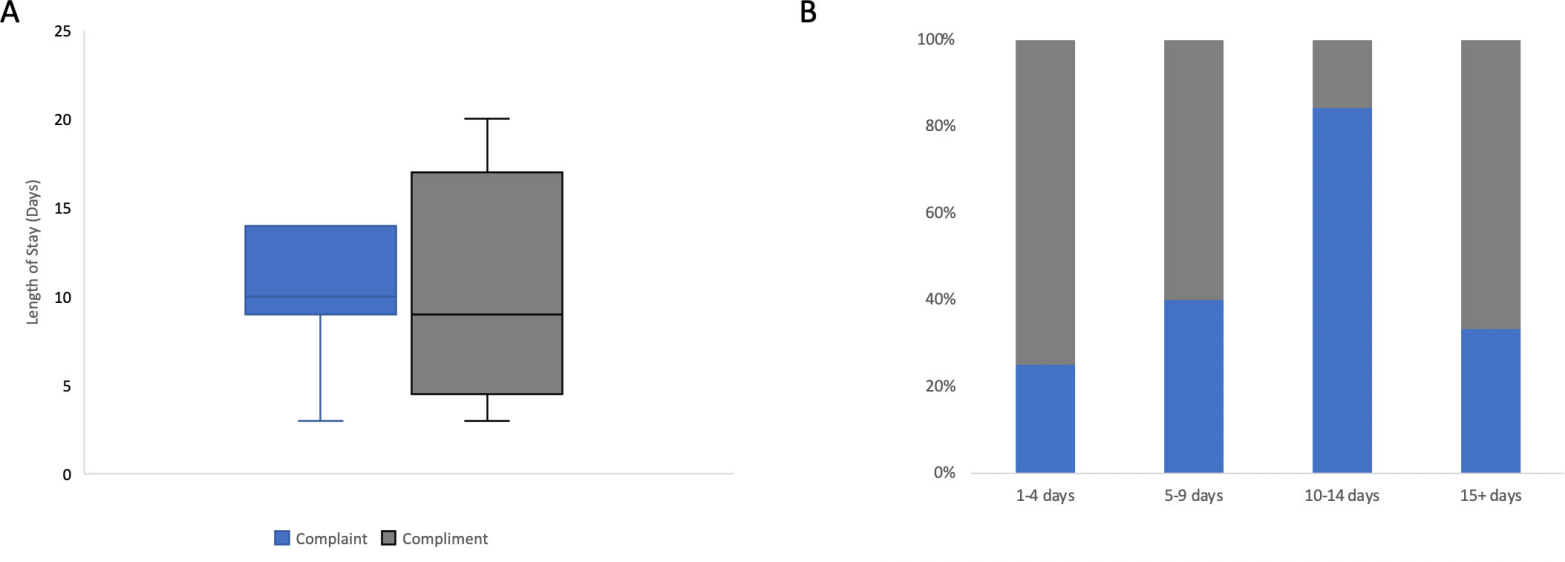
Complaints or compliments, and length of stay at tertiary centre. While overall not significant (A), there was a trend for complaints to become more likely/compliments less likely with length of stay up to 15 days (B). (A) Box and Whisker Plot of length of stay, with outliers removed. (B) Stacked bar chart showing the relative proportion of events per time window.

Although other factors were not significantly associated with complimenting, the rate of reoperation was lower among compliments (10%) than complaints (40%) and complaints appeared more common as the length of stay increased (figure 3).

### Comparison of cSDH with and without PALS interations

In order to explore how PALS data interacted with a more traditional and quantitative analysis of patient healthcare record data,^11^ these factors were also compared visually with a previous observational data set. Owing to its anonymised nature statistical comparison was not possible. This was performed for complaints (table 2) and compliments (table 3) separately. Both cases with complaints or compliments appeared less likely to be referred from another hospital, or suffer a complication, but more likely to stay over 10 days or be on anticoagulation before diagnosis. Median age, gender, and admission to operation length appeared broadly comparable.

**Table 2:** A comparison of compliment cases with all chronic subdural haematoma (cSDH) cases

Variables		Complaints (% of complaints)	All cSDH cases
N		15	531
Median age (IQR)		79 (72–85)	77 (69–84)
Gender	Male	11 (73)	376 (71%)
ASA	n with ASA ≥ 3	7 (47)	*271 (61%) (out of those recorded*)
Source of PALS interaction	Patient	3 (20)	
	Other	12 (80)	
Referred from other hospital	Yes	10 (67)	93% (491/530)
Length of stay	>10 days	11 (73)	137 (26%)
Admission to operation	>1 day	5 (33)	217 (40.2%)
Reoperation	Yes	6 (40)	
Anticoagulation	Yes	4 (27)	9.2% (49/530)
Complication (AKI or MI)	Yes	3 (20)	44% (239)

AKI, acute kidney injury; ASA, American Society of Anesthesiologists; MI, myocardial injury; PALS, Patient Advise and Liason Service.

**Table 3:** A comparison of complaint cases with all chronic subdural haematoma (cSDH) cases

Variables		Compliments (% of compliments)	All cSDH cases
N		10	531
Median age (IQR)		81 (68–87)	77(69-84)
Gender	Male	10 (100)	376 (71%)
ASA	n with ASA ≥ 3	7 (70)	*271 (61%) (out of those recorded*)
Source of PALS interaction	Patient	9 (90)	
	Other	1 (10)	
Referred from other hospital	Yes	6 (60)	93% (491/530)
Length of stay	>10 days	5 (50)	137 (26%)
Admission to operation	>1 day	3 (30)	217 (40.2%)
Reoperation	Yes	1 (10)	
Anticoagulation	Yes	3 (30)	9.2% (49/530)
Complication (AKI or MI)	Yes	2 (20)	44% (239)

AKI, acute kidney injury; ASA, American Society of Anesthesiologists; MI, myocardial injury; PALS, Patient Advise and Liason Service.

## Discussion

This is the first study to evaluate the use of PALS data within a tertiary service, specifically in neurosurgery using cSDH as an exemplar. cSDH was chosen as common condition, involving a regional pathway with a stereotyped presentation and treatment paradigm.

In this series of patients treated for a cSDH, 2% registered a compliment and 3% a complaint. Patients were more likely to register a compliment while family members a complaint. The HCAT was easily applied, with high inter-rater agreement. It was also able to highlight communication issues, of which 75% related to relative communication, which cannot be captured through traditional quantitative analysis of healthcare records.^11^ Nevertheless, PALS interactions mirrored some themes also highlighted using such an approaching, including anticoagulation use and length of stay. However, for a regional service it appeared to under-represent patients transferred from another hospital. This may relate to the geographical distances between relatives and the tertiary provider, although further research is required to explore such barriers.

### Added value of PALS data

While only a single pathology was studied and contextual factors will limit comparisons, it is noteworthy that the 3% incidence of complaints is considerably greater than previous general analyses which report an incidence of between 0.1% and 0.9%.^8 18^ Of note, the majority of complaints were coded by HCAT as communication issues, and submitted by relatives. While consistent with previous studies^19 20^ including medicolegal cases,^21^ online forums^22^ and national databases,^16^ this is significant, as communication issues are poorly identified from a traditional quantitative review of case records, and would almost certainly fail to capture the perspective of relatives. This is particularly important for informing care of patients which may have impaired mental capacity, a common scenario in neurological disorders.

Perspectives on communication are typically evaluated using qualitative studies such as surveys, interviews or focus groups, which are labour intensive, with poor scalability and subject to sampling bias. This highlights the potential for standardised analysis of PALS data to quantify a different aspect of healthcare provision.^23^

In this study, many issues highlighted related to changes in care plans, such as postponed or delayed surgery, and uncertainty over onward care following surgery, including repatriation. This would be compatible with a nationwide examination of complaints data, where a third of registered complaints occurred on the ‘boundaries’ of hospital care (at either admission or discharge).^16^

There are key challenges for effective communication within an emergency tertiary service. Care is delivered regionally alongside other, often more urgent, emergency workload. Moreover, the shift pattern introduced by ‘on call’ structures, offers another means by which care and continuity is further fragmented. Systems for repatriation are similarly disjointed with referrals often made to acute ‘on-take’ specialities who may or may not end up caring for the patient at the point of their ultimate repatriation. It is therefore important to have robust mechanisms for identifying service performance in these areas, which cannot be readily derived from quantitative record data.

Our findings therefore support using PALS interaction data for identifying areas for service improvement, particularly for cSDH, where patients are often elderly, with complex conditions and, due to the nature of the disease process, may have impaired capacity and communication issues that may be compounded by temporal and geographic obstacles in communication with relatives. This latter feature of cSDH care may also underpin the predominance of relative versus patient based complaints; a study of complaints among critical care patients found relatives were more likely to complain during their stay on critical care, but this reduced once the patient was on a ward.^24^ This could be attributed to the fact that some patients on critical care will have reduced mental capacity and may be too unwell to complain.

### Potential limitations

The value of PALS data to inform care has its criticisms, including a potential preference for ‘motivated’ individuals and therefore an under-estimation of overall care problems and potential skew on care themes. Additionally, the typically low aggregate incidence is a poor metric for assessing change.^6 7^ However, in this study, the higher incidence rate of 3% is more favourable for measuring change and based on the interaction of key variables with our previous study^11^ and those identified in the British Neurosurgical Trainee Audit,^12 13^ it appears PALS cases overlap with problems identified using traditional appraisal methodologies.

One of the key objectives for this study, was to consider the impact of PALS data, on a regional (tertiary) service. In this series the majority of cases submitting compliments or complaints were local to the tertiary centre. This suggests sole analysis of the tertiary centre’s PALS may fail to capture all complaints/compliments. While this could be solved through the acquisition of regional PALS data, there are potential hurdles for data sharing agreements and additionally, as the logging of PALS data are non-standard, their interpretation without access to local case notes. That said, it is unclear whether this would have additional benefit; in this series, key complaint themes reached saturation, including communication and around timing of treatment or transfer, providing key targets for service improvement. Moreover, regional patients were represented.

Further our application of the HCAT tool here showed excellent agreement, with the exception of a case raising concerns about care at a regional hospital (the only such example); one reviewer coded this as clinical management, whereas another as an institutional management problem. This may indicate a requirement for the HCAT criteria to be adapted to handle regional concerns.

## Conclusion

While there are some limitations for its application to a tertiary service which may benefit from further development, PALS data are a simple and routinely collected resource able to measure and provide unique perspectives on care, such as communication. It is readily suited to quality improvement and audit initiatives.

## Data Availability

Data are available upon reasonable request. Anonymous dataset available on request.
