# Supplementary material for: Can compliment and complaint data inform the care of individuals with chronic subdural haematoma (cSDH)?
Source: BMJ Open Qual. 2021 Sep 16;10(3):e001246. doi: 10.1136/bmjoq-2020-001246 (PMC8451295; doi:10.1136/bmjoq-2020-001246)
Supplement: Supplementary data [file bmjoq-2020-001246supp001.pdf]

Supplementary 1: A summary of the PALS cases in this study.

PALS Interaction	Complainant	Primary HCAT Code	Case Summary	HCAT Severity	HCAT Harm Coding
**Complaint**	Relative	Management	Daughter unhappy father's surgery delayed (it was postponed)	Low	None
**Complaint**	Relative	Management	Patient's wife concerned about repatriation to local hospital due to concerns about care they had previously received there. Wanted more medical information about surgery and current condition.	Low	None
**Complaint**	Relative	Relative behaviour	Son continued to email PALS frequently. Staff raised concerns that son was filming on the ward.	Low	None
**Complaint**	Patient	Management	Property could not be found after said items were stored in the hospital's safe.	Low	None
**Complaint**	Relative	Relationship	Husband questioned why wife no longer for surgery when they were transferred for that reason.	Low	None
**Complaint**	Relative	Relationship	Son concerned father not safe for discharged despite SAFE team opinion.	Low	None
**Complaint**	Relative	Management	Daughter claimed harm by delaying elective surgery in favour of emergency case.	Low	None
**Complaint**	Relative	Management	Father had a fall and hit head on sink next to bed. Daughter thought sink too close.	Medium	Minimal
**Complaint**	Patient	Relationship	Felt ignored and had three hour wait for laxatives after operation.	Medium	None
**Complaint**	Relative	Relationship	Daughter thought discharge was too early. Lack of information regarding community care/support available and felt fall at home was due to hospital discharge.	Medium	None
**Complaint**	Relative	Relationship	Daughter raised concerns about transfer to another hospital without telling family.	Medium	None
**Complaint**	Staff	Management	Transfer to district hospital without belongings.	Medium	None

**Complaint**	Relative	Relationship	Husband perceived poor communication. Staff provided incorrect information regarding wife's location which caused delay to treatment and discharge.	Medium	None
**Complaint**	Relative	Relationship	Wife perceived delayed surgery. Lack of information about condition.	Medium	None
**Complaint**	Patient	Relationship	Unhappy with discharge arrangements.	Medium	None
**Compliment**	Relative	Clinical	Compliment of care.		
**Compliment**	Patient	Relationship	Compliment of care, especially kindness shown by a staff nurse		
**Compliment**	Patient	Clinical	Compliment of care		
**Compliment**	Patient	Clinical	Compliment of care		
**Compliment**	Patient	Clinical	Compliment of care and treatment.		
**Compliment**	Patient	Clinical	Compliment of care		
**Compliment**	Patient	Clinical	Compliment of care		
**Compliment**	Patient	Clinical	Complimented care and treatment		
**Compliment**	Patient	Clinical	Compliment of care		
**Compliment**	Patient	Clinical	Compliment of care		
