# Supplementary figures and images for: Can compliment and complaint data inform the care of individuals with chronic subdural haematoma (cSDH)?
Source: BMJ Open Qual. 2021 Sep 16;10(3):e001246. doi: 10.1136/bmjoq-2020-001246 (PMC8451295; doi:10.1136/bmjoq-2020-001246)

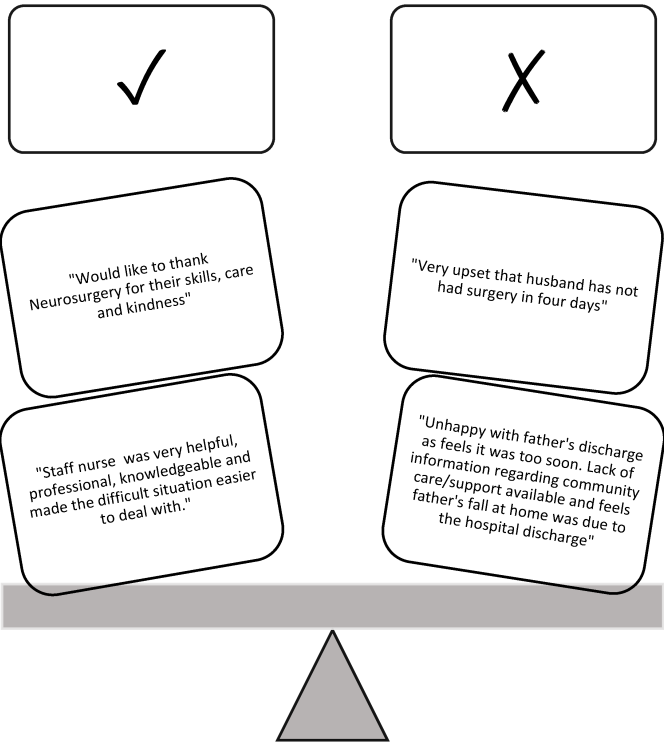

Supplementary 2: Some example quotes from a selection of compliments and complaints.

Supplement: Supplementary data [file bmjoq-2020-001246supp002.pdf]
